# Assessment of T1rho relaxation times after reperfused myocardial infarction

**DOI:** 10.1186/1532-429X-18-S1-W13

**Published:** 2016-01-27

**Authors:** Marie Madden, Shahid Mohammed, Francisco Contijoch, James J Pilla, Joseph H Gorman, Yuchi Han, Robert C Gorman, Walter R Witschey

**Affiliations:** 1 Surgery, University of Pennsylvania, Philadelphia, PA USA; 2 Radiology, University of Pennsylvania, Philadelphia, PA USA; 3 Medicine, University of Pennsylvania, Philadelphia, PA USA

## Background

T1ρ MRI is an emerging method for high spatial resolution tissue characterization of myocardial infarct (MI), but the evolution of T1ρ in the first month after reperfused MI is uncertain. We conducted a study of reperfused MI in pigs to serially monitor T1ρ relaxation times at baseline, 1 and 4 weeks post-MI and correlated these results with T2, native T1, and histological findings.

## Methods

13 pigs underwent 90 minutes of occlusion of the circumflex artery branches. T1ρ, T2 and native T1 MRI data were collected at terminal 1 week (n = 6) and 4 weeks (n = 7) post-MI studies. 2D T1ρ single-shot balanced steady-state free precession (bSSFP) sequences were performed using a spin echo, spin lock T1ρ pulse cluster (90_x_ - SL_y_ - 180_y_ - SL_-y_ - 90_-x_) with the following parameters: TSL = 2-50 ms, B_1_ = 500 Hz. T2 MRI was obtained 90_x_ - 180_y_ - 90_-x_ using the same readout parameters. T1 maps were obtained with a modified Look-Locker sequence (MOLLI WIP sequence #448B, 5-3-3, Siemens), utilizing a single-shot acquisition with 8 inversion times. For LGE MRI, the animal received 0.1 mmol/kg intravenous injection of gadolinium contrast (MultiHance). Late-gadolinium enhanced (LGE) MRI was obtained using a 3D multishot phase-sensitive inversion recovery (PSIR) sequence with bSSFP spatial encoding. *Ex vivo* MRI and histology was performed at each terminal time point. Fibrotic tissue was assessed with trichrome staining. *In vivo* MRI was spatially correlated with *ex vivo* imaging and histopathology.

## Results

Infarct T1ρ increased 39.0 (1 week) and 32.1 ms (4 weeks) and T2 increased 21.0 ms (1 week) and 16.2 (4 weeks) after infarction. *In vivo* T1ρ was longer than T2 in normal (baseline), remote and infarcted tissue at 1 and 4 weeks (p < 0.05). Native T1 increased 288.0 and 212.4 ms at 1 and 4 weeks post-infarction (p < 0.05). The percent increase in T1ρ, T2 and T1 from baseline was 76, 48 and 24% at 1 week and 56, 36 and 21% at 4 weeks in the infarct region. T1ρ post-infarction area at 1 week was highly correlated with infarct fibrosis (p < 0.05).

## Conclusions

T1ρ relaxation times were highly correlated with alterations in post-MI pathology at 1 and 4 weeks and therefore it may be a useful method for non-contrast enhanced imaging of infarction.

**Figure 1:**
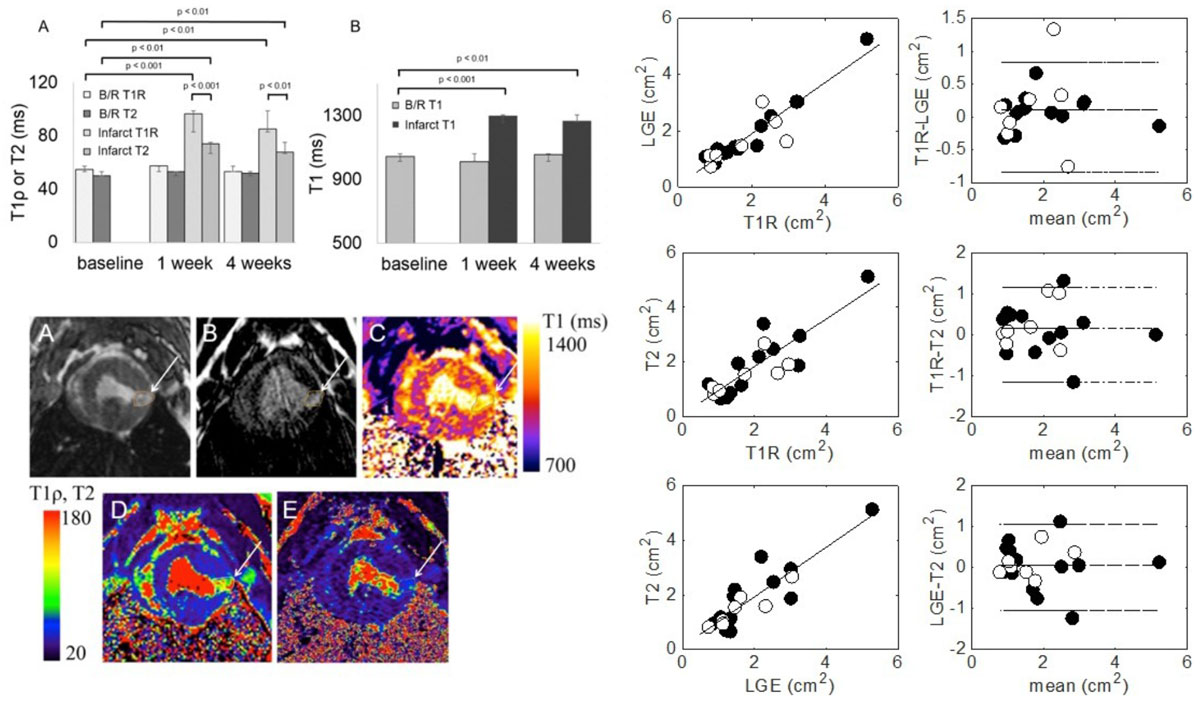
Figure 1

